# Neighbor danger: Yellow fever virus epizootics in urban and urban-rural transition areas of Minas Gerais state, during 2017-2018 yellow fever outbreaks in Brazil

**DOI:** 10.1371/journal.pntd.0008658

**Published:** 2020-10-05

**Authors:** Lívia Sacchetto, Natalia Ingrid Oliveira Silva, Izabela Maurício de Rezende, Matheus Soares Arruda, Thais Alkifeles Costa, Érica Munhoz de Mello, Gabriela Fernanda Garcia Oliveira, Pedro Augusto Alves, Vítor Emídio de Mendonça, Rodolfo German Antonelli Vidal Stumpp, Alaine Izabela Alves Prado, Adriano Pereira Paglia, Fernando Araújo Perini, Maurício Lacerda Nogueira, Erna Geessien Kroon, Benoit de Thoisy, Giliane de Souza Trindade, Betânia Paiva Drumond

**Affiliations:** 1 Department of Microbiology, Universidade Federal de Minas Gerais, Belo Horizonte, Minas Gerais, Brazil; 2 Centro de Controle de Zoonoses, Belo Horizonte, Minas Gerais, Brazil; 3 Instituto René Rachou/Fundação Oswaldo Cruz Minas, Belo Horizonte, Minas Gerais, Brazil; 4 Department of Zoology, Universidade Federal de Minas Gerais, Belo Horizonte, Minas Gerais, Brazil; 5 Department of Genetics, Ecology and Evolution, Universidade Federal de Minas Gerais, Belo Horizonte, Minas Gerais, Brazil; 6 Department of Dermatological, Infectious and Parasitic Diseases, Faculdade de Medicina de São José do Rio Preto, São José do Rio Preto, São Paulo, Brazil; 7 Institut Pasteur de la Guyane, Cayenne, French Guiana; Fundacao Oswaldo Cruz, BRAZIL

## Abstract

**Background:**

From the end of 2016 until the beginning of 2019, Brazil faced a massive sylvatic yellow fever (YF) outbreak. The 2016–2019 YF epidemics affected densely populated areas, especially the Southeast region, causing thousands of deaths of humans and non-human primates (NHP).

**Methodology/Principal findings:**

We conducted a molecular investigation of yellow fever virus (YFV) RNA in 781 NHP carcasses collected in the urban, urban-rural interface, and rural areas of Minas Gerais state, from January 2017 to December 2018. Samples were analyzed according to the period of sampling, NHP genera, sampling areas, and sampling areas/NHP genera to compare the proportions of YFV-positive carcasses and the estimated YFV genomic loads. YFV infection was confirmed in 38.1% of NHP carcasses (including specimens of the genera *Alouatta*, *Callicebus*, *Callithrix*, and *Sapajus*), from the urban, urban-rural interface, and rural areas. YFV RNA detection was positively associated with epidemic periods (especially from December to March) and the rural environment. Higher median viral genomic loads (one million times) were estimated in carcasses collected in rural areas compared to urban ones.

**Conclusions/Significance:**

The results showed the wide occurrence of YF in Minas Gerais in epidemic and non-epidemic periods. According to the sylvatic pattern of YF, a gradient of viral dissemination from rural towards urban areas was observed. A high YF positivity was observed for NHP carcasses collected in urban areas with a widespread occurrence in 67 municipalities of Minas Gerais, including large urban centers. Although there was no documented case of urban/*Aedes* YFV transmission to humans in Brazil during the 2016–2019 outbreaks, YFV-infected NHP in urban areas with high infestation by *Aedes aegypti* poses risks for YFV urban/*Aedes* transmission and urbanization.

## Author summary

Brazil faced the most massive sylvatic yellow fever (YF) outbreak in 2016–2019. The outbreak affected highly densely populated areas, and Minas Gerais was the most affected state with thousands of deaths of human and non-human primates (NHP). We investigated the yellow fever virus (YFV) RNA in NHP carcasses collected throughout Minas Gerais in 2017 and 2018. We demonstrated the wide occurrence of YFV-infected NHP, including the viral persistence during the non-epidemic dry season of 2017. YFV RNA was detected in NHP carcasses in the urban, urban-rural interface and rural areas. We have also detected new YF cases in 49 municipalities where YF cases have not been previously detected during the outbreaks. Estimates of YFV genomic load in naturally infected NHP carcasses showed high and similar loads in specimens (*Alouatta*, *Callithrix*, and *Callicebus*) collected in rural areas and lower genomic loads in the urban-rural interface and urban *Callithrix* specimens. The presence of YFV inside urban areas poses an imminent risk, although no human case was epidemiologically linked to urban/*Aedes* transmission during the last outbreaks in Brazil.

## Introduction

Yellow fever virus (YFV) (family *Flaviviridae*, genus *Flavivirus*) is endemic in tropical and subtropical regions of Africa, Central and South America. Yellow fever (YF) presents a broad spectrum of severity, with clinical manifestations ranging from self-limited febrile to fatal disease in humans [1,2]. There is no specific treatment for YF, and vaccination is recommended for people who live in or travel to at-risk areas [2].

Urban YF, transmitted by *Aedes aegypti* vector, was a severe threat to Brazil’s human health until 1942 when the last case was registered in Acre state [1]. Nowadays, the virus is maintained in the sylvatic cycle involving Neotropical non-human primates (NHP) and sylvatic mosquitoes (*Haemagogus* spp. and *Sabethes* spp.) [2]. Although these mosquitoes mainly feed on NHP, they can incidentally transmit the virus to humans [2]. The reemergence of sylvatic YF outside the Brazilian Amazon basin has been reported, especially during the rainy season, called the epidemic period [3].

Since the eradication of urban YF, Brazil faced the largest sylvatic YF outbreak from the end of 2016 until the beginning of 2019 [4]. YFV was introduced into Southeast [4–8], from the Midwest region [8] spreading southward, causing thousands of NHP and human cases and deaths [4,9–11]. From July 2016 until June 2019, 2,240 human cases and 760 deaths were confirmed, mostly in the Southeast region (99.2%), and over 2,590 YF epizootics were confirmed in Brazil [4,10–13]. Minas Gerais, Southeast Brazil, was one of the most affected states during the YF outbreaks. Between July 2016 and June 2018, the Secretary of Health of Minas Gerais confirmed 1,002 human cases and 340 deaths, and more than 490 epizootics in NHP [14]. Minas Gerais is in the recommended area for YF vaccination since 2008, but until the end of 2016, the state presented low average vaccination coverage (57.26%), reaching 92.71% by the end of 2018 [15].

The rapid spread and the extent of the latest recent YF outbreaks in the Southeast region, including highly densely populated urban centers in the states of Minas Gerais, São Paulo, Espírito Santo, and Rio de Janeiro [10,11], have raised concerns regarding YFV reintroduction in epidemiologically receptive areas, with millions of naïve people for YFV. NHP is the primary host of the YF sylvatic cycle, and even though NHP are mostly observed in rural/sylvatic zones, these animals are commonly found in urban-rural transition and urban areas in Brazil. Given the high numbers of epizootics during YF outbreaks, this study aimed at the molecular investigation of YFV in NHP from Minas Gerais. Our results showed the widespread occurrence of YFV in Minas Gerais and, hastily, YFV in NHP carcasses collected inside urban matrixes and urban-rural transition areas, including large urban centers.

## Material and methods

### Study area and samples

**Fig 1:**
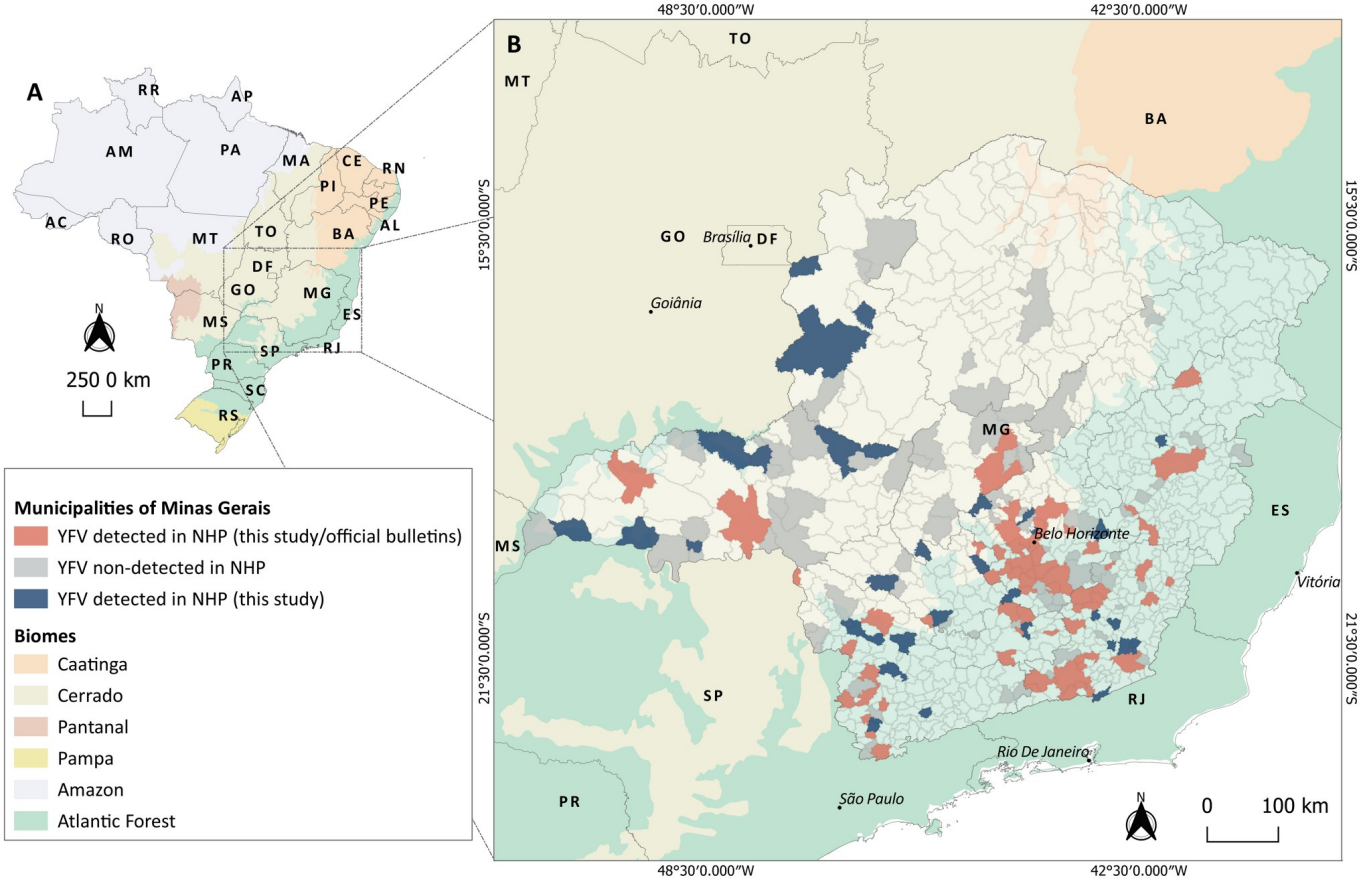
Yellow fever virus (YFV) investigation in non-human primate (NHP) carcasses, in Minas Gerais state, January 2017-December 2018. (A) Geopolitical map of Brazil with biomes distribution. (B) Map of Minas Gerais state indicating municipalities from where NHP carcasses were collected and investigated for YFV RNA. Municipalities are colored as follows: in grey are 120 municipalities without detection of YFV in NHP (in this study); in light red are 110 municipalities with detection of YFV in NHP (this study and official bulletins); and in blue are 49 municipalities with detection of YFV in NHP (only in this study) where YFV circulation has not been described, during 2016–2018 outbreaks. Based on official bulletins, YFV was detected (humans or NHP) in another 31 municipalities of Minas Gerais by the State Surveillance Secretary during 2016–2018 outbreaks [13,21,22]. Abbreviations referring to Federal District and states as follows: AC: Acre; AL: Alagoas; AP: Amapá; AM: Amazonas; BA: Bahia; CE: Ceará; DF: Distrito Federal; ES: Espírito Santo; GO: Goiás; MA: Maranhão; MT: Mato Grosso; MS: Mato Grosso do Sul; MG: Minas Gerais; PA: Pará; PB: Paraíba; PR: Paraná; PE: Pernambuco; PI: Piauí; RR: Roraima; RO: Rondônia; RJ: Rio de Janeiro; RN: Rio Grande do Norte; RS: Rio Grande do Sul; SC: Santa Catarina; SP: São Paulo; SE: Sergipe; and TO: Tocantins. Numbers indicate the mesoregions of Minas Gerais as follows: 1. Triângulo Mineiro e Alto Paranaíba; 2. Northwest; 3. North; 4. Jequitinhonha; 5. Vale do Mucuri; 6. Vale do Rio Doce; 7. Zona da Mata; 8. South/Southwest; 9. West; 10. Central; 11. Metropolitan region; 12. Campo das Vertentes. Maps were created using the QGIS software version 3.8.2.

During YF outbreaks in Brazil (January 2017 to December 2018), we analyzed 771 liver samples from carcasses of free-living NHP from different areas of Minas Gerais state. Data regarding ten NHP carcasses previously screened for the YFV RNA [7] were included in data analysis, totalizing 781 individuals analyzed here. The NHP carcasses consisted of a convenient sampling, collected in the context of the Brazilian Yellow Fever Surveillance Program [16], of epizootics that occurred in 2017 and 2018. Since biological samples were obtained from carcasses, blood sampling was not possible, and liver samples were selected for YFV investigation, due to the hepatotropic of YFV in humans and NHP [1,4, 16, 17]. Minas Gerais is in Southeast Brazil (Fig 1), with 586,521.121 km^2^ and 21,040,662 inhabitants. The state has 853 municipalities grouped into 12 mesoregions. Three main biomes cover the state: Cerrado, Atlantic Forest, and Caatinga [18] (Fig 1).

The NHP carcasses were collected in the field by health surveillance agents (Brazilian Yellow Fever Surveillance Program [16], frozen (-20 °C), and transported to the Laboratory of Zoonosis of Belo Horizonte/Minas Gerais (LZOON-BH). At LZOON-BH, NHP carcasses were defrosted and visually inspected regarding the stage of decomposition, taking into account different tissue aspects: (*i*) brightness (bright or opaque); (*ii*) color (regular aspect, greenish, greyish or whitish); (*iii*) consistency (if the tissue was firm or softened); (*iv*) smell (whether the carcass was smelling rotten or not); and (*v*) presence or absence of parasites (larvae). Based on that, liver samples were categorized according to their preservation status: good (n = 465; 59.5%), intermediate (n = 211; 27.0%), or bad (n = 59; 7.5%). After defrosting, liver fragments (0.5 cm) were collected from the carcasses and placed into microtubes containing RNA later solution (Ambion, USA) by the LZOON team. The microtubes were kept at 4 °C for 16 hours and then stored at -20 °C until used for RNA extraction at Laboratório de Vírus/Universidade Federal de Minas Gerais (UFMG).

Using the information on the address or geographic coordinates where carcasses were collected, each carcass was classified based on land use: urban (built-up) areas, urban-rural transition areas, and rural/sylvatic areas. Urban areas (built-up) corresponded to cities, towns, and villages or isolated urban areas, characterized by transformations resulting from urban development (e.g., paved streets, electric lighting, sewerage, buildings, and intense human occupation) [19]. A buffer zone (2.0 kilometers) was established on the outskirts of urban areas, delineating an urban-rural transition area (interface). The rural/sylvatic areas covered the entire area located outside these limits. Each localization of carcasses was checked using satellite imagery (Google Maps, available at https://www.google.com/maps) to determine the boundaries of the urban areas [19] urban-rural interface areas and rural/sylvatic areas as stated above. NHP carcasses analyzed here were collected in Cerrado and Atlantic Forest areas, but not in Caatinga regions.

NHP carcasses were identified at species or genus level, by the Veterinary team at LZOON or by the Zoology/Mastozoology teams at UFMG, using morphological criteria [20–22]. Out of 781 NHP analyzed in this study, 360 carcasses (collected in 2017) were forward to UFMG laboratories, and some of them were taxidermized and deposited in the Center of Taxonomic Collections of Universidade Federal de Minas Gerais (CCT-UFMG), Brazil [20–22]. The Committee on Ethics in the Use of Animals of UFMG approved the study (CEUA 98/2017).

### YFV molecular investigation

Total RNA was extracted from approximately 30 mg of the liver preserved in RNA later solution (Ambion, USA), using RNeasy Minikit (Qiagen, USA). RNA extraction was performed in batches of 11 samples plus one negative control (nuclease-free water). RNA samples were submitted to a one-step real-time polymerase chain reaction (RT-qPCR), with primers targeting the gene coding for β-actin [23], using GoTaq 1-Step RT-qPCR System (Promega, USA). All samples were positive, showing the suitability of samples for RT-qPCR. RNA samples and the negative extraction controls were screened in duplicate for YFV RNA using RT-qPCR [24]. Non-template (nuclease-free water) and YFV 17DD RNA (provided by Bio-Manguinhos/FIOCRUZ) were used as negative and positive controls, respectively. Samples were considered positive when they presented amplification in duplicate, considering the threshold for cycle quantification value (Cq) of 37. Since between the range of 37–40 Cq indicate minimal quantities of DNA, with low confidence for the results, Cqs>37 or undetermined were considered negative. The results of RT-qPCR runs were manually inspected for the correction of baseline and threshold parameters whenever necessary due to heterogeneity in the amount of input RNA among different samples.

The Cqs are inversely proportional to the input target nucleic acid in the sample, which could indirectly reflect the YFV genomic load in the samples. In that way, we performed a quantitative RT-qPCR (Bio Gene Research Febre Amarela PCR kit—Bioclin-Quibasa, Brazil) using RNA obtained from 28 YFV-positive samples to have an estimative of YFV genomic load in the liver. We determined the number of genomic copies per gram (gc/g) of the liver, and we estimated the genomic loads in all YFV-positive carcasses using linear regression and the observed Cqs.

From 11 YFV-positive samples, a partial sequence of the envelope gene (primers 5-left and 6-right) was obtained [5], using the GoTaq Hot Start Colorless Master Mix (Promega, USA). The amplicons were purified and sequenced with the ABI3130 platform (Applied Biosystems, USA). The consensus sequences were generated (Geneious v.9.1.6—Biomatters, NZL; https://www.geneious.com/) and used for phylogenetic reconstruction, using the maximum likelihood method implemented in MEGA7 (https://www.megasoftware.net/). The final tree was edited and visualized in FigTree v.1.4.4 (http://tree.bio.ed.ac.uk/software/figtree/).

### Statistical analyses

For statistical analyses, samples were subdivided into different groups according to the period of sampling: (*i*) epidemic (December-May) and non-epidemic (June-November) periods; (*ii*) bimester within epidemic (December/January, February/March, and April/May) and within non-epidemic periods (June/July, August/September, and October/November); (*iii*) NHP genera: *Callithrix*, *Callicebus*, and *Alouatta* (*Sapajus* was not included due to the low number of specimens); (*iv*) environment: rural, urban-rural interface, and rural areas; and (*v*) environment together with NHP genera: urban *Callithrix*, urban-rural *Callithrix*, rural *Callithrix*, rural *Callicebus*, and rural *Alouatta* (given the low number of specimens, *Alouatta* and *Callicebu*s from urban and urban-rural interface were not included in the analyses). The comparison of proportions of YFV-positive carcasses within groups was performed using chi-square and chi-square for trend. The Kruskal-Wallis test and multiple pairwise-comparisons between groups by Wilcoxon rank-sum test were used to compare the (median) Cq values observed for the positive samples within each group. Median Cq values were compared only using data from carcasses in good and intermediate conservation status (see Results). Statistical analyses were run in R v.3.6.0 (https://www.r-project.org/) and Epitools (http://epitools.ausvet.com.au/). Bonferroni correction for multiple comparisons was applied, with differences considered statistically significant at p< 0.0125.

## Results

We analyzed data from 781 NHP carcasses collected from January 2017 to December 2018 (2017: 50.8%, and 2018: 49.1%), during the epidemic (75.8%) and non-epidemic periods (24.2%), in urban (57.7%), urban-rural interface (6.9%), and rural areas (35.3%) (S1 Fig). The identified NHP carcasses belonged to different families and genera: Atelidae (*Alouatta guariba*, *Alouatta caraya*, and *Alouatta* sp.) (6.6%); Pitheciidae (*Callicebus personatus*, *Callicebus nigrifrons*, and *Callicebus* sp.) (4.1%); Cebidae (*Sapajus* sp. *and Sapajus nigritus*) (0.2%); and Callitrichidae (*Callithrix geoffroyi*, *Callithrix aurita*, *Callithrix penicillata*, *Callithrix* sp., and hybrid marmosets) (84.3%) (Table 1).

**Table 1:** Non-human primate carcasses tested for yellow fever virus RNA.

Genera/species	Total carcasses		YFV-positive carcasses	
	Total	Total by genus (%)	Total	Total by genus (%)
*Alouatta caraya*	2		1	
*Alouatta guariba*	17		12	
*Alouatta* sp.	33	52 (6.66%)	28	41 (13.76%)
*Callicebus nigrifrons*	19		12	
*Callicebus personatus*	7		6	
*Callicebus* sp.	6	32 (4.10%)	4	22 (7.38%)
*Callithrix aurita*	2		1	
*Callithrix geoffroyi*	21		10	
*Callithrix penicillata*	170		44	
*Callithrix* sp.	466	659 (84.38%)	155	210 (70.47%)
*Sapajus nigritus*	1		1	
*Sapajus* sp.	1	2 (0.26%)	0	1 (0.34%)
Non-ID	36	36 (4.61%)	24	24 (8.05%)
Total	781		298	

YFV: yellow fever virus. Liver samples of non-human primate carcasses collected in Minas Gerais state, Brazil (Jan 2017-Dec 2018) were tested for YFV RNA using the RT-qPCR [24].

YFV infection was confirmed in 298 out of 781 carcasses (38.1%, 95% CI = 34.7–41.6%). Regardless the preservation status of the carcasses, all samples were analyzed, and YFV RNA was detected in the liver of carcasses in bad (16/59, 27.1%, 95% CI = 17.4–39.6%), intermediate (85/211, 40.2%, 95% CI = 33.9–47.0%) or good (187/465, 40.2%, 95% CI = 35.6–44.7%) preservation status. No association between the preservation status of carcasses and YFV RNA detection was observed (*x*^*2*^ = 3.91, p = 0.14).

**Fig 2:**
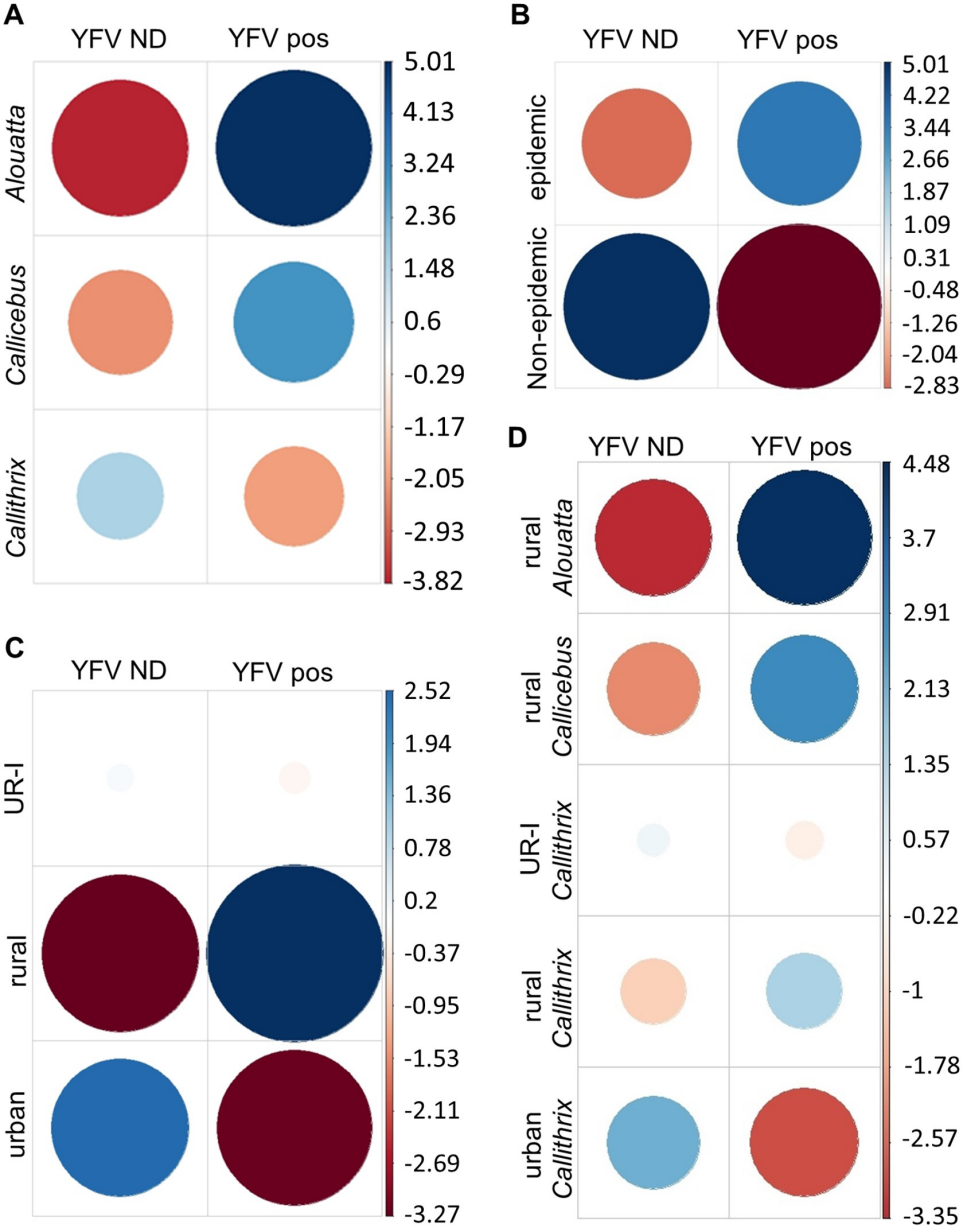
Association between detection of yellow fever virus (YFV) in the liver of non-human primate (NHP) carcasses with (a) NHP genera, (b) period of sampling (c) environment, and (d) environment plus NHP genera. YFV ND: yellow fever virus RNA was not detected. YFV pos: yellow fever virus RNA was detected. Pearson residuals (standardized) were extracted from the chi-square function and plotted. In each cell, the size of the circle is proportional to the amount of cell contribution, and colors indicate positive residuals (blue) or negative residuals (red). The vertical bars indicate Pearson residuals values. Analyses were run in R software v.3.6.0.

YFV RNA was detected in specimens of *Alouatta*, *Callicebus*, *Callithrix* (Table 1), and *Sapajus*. The investigation of the association between the positivity and NHP genera considered only data regarding *Callithrix*, *Callicebus*, and *Alouatta* (n = 743). An association between the YFV positivity and NHP genera (*x*^*2*^ = 60.51, p <0.0001) was observed, with a stronger positive association between the detection of YFV RNA and specimens of *Alouatta* (78.8%) and *Callicebus* (68.7%) compared to *Callithrix* (31.8%) (Fig 2A, Table 2). No difference in positivity for YFV was observed between *Alouatta* and *Callicebus* (*x*^*2*^ = 0.60, p = 0.44), and a higher odds ratio (OR) for YFV detection was observed for both genera (OR = 7.96 and OR = 4.70, respectively), compared to *Callithrix* (Table 2).

**Table 2:** Molecular detection of yellow fever virus RNA in non-human primate carcasses.

Variable	Pos/total	Positivity % (95%CI)	OR (95%CI)	p-value*
**Genera**^**1**^				
*Callithrix*	210/659	31.87 (28.42–35.52%)		
*Callicebus*	22/32	68.75 (51.43–82.05%)	4.70 (2.19–10.11)	<0.0001
*Alouatta*	41/52	78.85 (65.97–87.76%)	7.96 (4.10–15.81)	<0.0001
**Period**^**2**^				
Non-epidemic	18/189	9.52 (6.12–14.50%)		
Epidemic	280/592	47.30 (43.31–51.32)	8.52 (5.11–14.22)	<0.0001
**Environment**^**3**^				
Urban	130/452	28.76 (24.60–32.90%)		
Urban-rural interface	19/53	35.85 (22.90–48.80%)	1.40 (0.78–2.52)	<0.0001
Rural	148/276	53.62 (47.73–59.42%)	2.85 (2.08–3.90)	<0.0001
**Environment** **/genera**^**4**^				
Urban *Callithrix*	120/436	27.52 (23.30–31.70%)		
Urban-rural *Callithrix*	16/48	33.33 (20.00–46.70%)	1.27 (0.67–2.39)	<0.0001
Rural *Callithrix*	74/175	42.29 (35.21–49.69%)	1.92 (1.33–2.77)	<0.0001
Rural *Callicebus*	18/26	69.23 (50.01–83.50%)	5.90 (2.50–13.94)	<0.0001
Rural *Alouatta*	33/43	76.74 (62.26–86.85%)	8.66 (4.14–18.12)	<0.0001

* 95% CI: 95% confidence interval. OR: odds ratio. Pos: yellow fever virus genome detected by RT-qPCR. ^1^The investigation of the association between the positivity and NHP genera considered only data regarding *Callithrix*, *Callicebus*, and *Alouatta* (n = 743, excluding non-identified specimens and one specimen of *Sapajus*). ^2,3^The investigation of the association between the positivity and ^2^the period of sampling (epidemic or non-epidemic) and ^3^the investigation of the association between the positivity and the environment (urban, urban-rural interface, and rural/sylvatic areas) included all specimens (n = 781). ^4^The investigation of the association between the positivity and the environment together with NHP genera included part of specimens (n = 728, non-identified specimens; specimens of *Sapajus*; and specimens of *Alouatta* and *Callicebu*s from urban and urban-rural interface were not included in the analyses). Liver samples of non-human primate carcasses collected in Minas Gerais state, Brazil (Jan 2017-Dec 2018), were tested for yellow fever virus RNA, using the RT-qPCR [24]. *Chi-square test with Bonferroni correction for multiple comparisons with differences considered statistically significant at p<0.0125. Analyses were run in R software v.3.6.0.

The detection of YFV RNA was negatively associated with the non-epidemic periods while positively associated with epidemic periods (*x*^*2*^ = 85.03, p< 0.0001) (Fig 2B), especially the first two bimesters of the epidemic period (Dec/Jan and Feb/Mar) (S2 Fig). An extremely high OR (OR = 14.63, 95% CI = 5.80–36.91, p< 0.0001) was observed for YFV detection in December /January followed by February/March (OR = 14.63, 95% CI = 2.00–12.35, p< 0.0001). The detection of YFV RNA was negatively associated with the last bimester of the epidemic period (Apr/May) (S2 Fig). No difference was observed between the proportions of YFV-infected carcasses collected in 2017 (n = 155/397; 39.0%, 95% CI = 34.3–43.9%) and 2018 (n = 126/384; 32.8%; 95% CI = 28.3–37.6%) (*x*^2^ = 0.19, p = 0.65). Throughout 2017, YFV was detected with a higher proportion of YFV-infected carcasses during the epidemic period (*x*^2^ = 17.37, OR = 3.28, 95% CI = 1.87–5.77, p< 0.001). In 2018, YFV was detected only in the epidemic period (S1 Fig).

NHP carcasses were collected in 279 municipalities located in 12 mesoregions of Minas Gerais state, covered by Cerrado and Atlantic Forest. YFV was detected in 159 municipalities (11 mesoregions) (Fig 1B), within urban (n = 67), urban-rural interface (n = 20), and rural (n = 106) environments. In 2017 and 2018, the highest numbers of YFV-positive carcasses were from Metropolitan (42.6%), Zona da Mata (51.0%), and the South/Southwest (43.9%) regions (S1 Table).

Regarding the environment, differences were observed among the proportions of YFV-positive carcasses collected in urban (28.7%) urban-rural interface (35.8%), and rural areas (53.6%) (*x*^*2*^ = 44.65, p< 0.0001) (Table 2). Analyses indicated a linear trend (*x*^*2*^ = 44.27, p< 0.0001) and higher odds ratio for detection of YFV from urban towards urban-rural interface (OR = 1.40) and rural areas (OR = 2.85) (Table 2). The detection of YFV had a strong positive association with the rural environment and a strong negative association with the urban environment (Fig 2C).

Next, we detected an association of YFV-positive carcasses with the environment and NHP genera altogether (*x*^*2*^ = 60.51, p< 0.0001) (Fig 2D). The proportions of YFV-positive carcasses of rural *Alouatta* (76.7%), rural *Callicebus* (69.2%), and rural *Callithrix* (42.2%) were higher than urban-rural *Callithrix* (33.3%) (*x*^*2*^≥ 7.70, p≤ 0.005) and urban *Callithrix* (27.5%) (*x*^*2*^≥ 18.34, p≤ 0.0001). A difference between the proportion of urban and rural YFV-infected *Callithrix* was observed (*x*^*2*^ = 11.76, p< 0.001), but no difference was observed between rural and urban-rural *Callithrix* (*x*^*2*^ = 2.78, p = 0.95) nor between urban and urban-rural *Callithrix* (*x*^*2*^ = 1.10, p = 0.29). Lower linear trend (*x*^*2*^ = 60.15, p< 0.0001) and odds ratio for YFV detection were observed, from rural *Alouatta* (OR = 8.66), rural *Callicebus* (OR = 5.90) and rural *Callithrix* (OR = 1.92) to urban-rural *Callithrix* (OR = 1.27) and finally urban *Callithrix* (Table 2).

Since Cqs reflect the YFV genomic load in liver samples, we analyzed the median Cq values for YFV-positive samples. There was no difference in median Cqs observed in carcasses in good versus intermediate conservation status (*x*^*2*^ = 9.08, p< 0.010), carcasses in bad conservation status were discarded from this analysis, and only results obtained from carcasses in good and intermediate conservation status were considered. A negative linear regression was observed, and a significant equation was obtained [10E (-0.2986 x Cq + 13.5094)] (R^2^ = 0.99 and p< 2.2E-16) to estimate the YFV genomic load using the Cqs in liver samples under the experimental conditions used here.

The median Cqs differed regarding the environment where carcasses were collected (*x*^*2*^ = 57.19, p< 0.0001) (Table 3). Lower median Cqs (higher estimated YFV genomic loads) were observed for carcasses collected in rural areas (median Cq = 13.0) compared to carcasses from urban-rural interface (median Cq = 31.5) (Table 3), and urban areas (median Cq = 32.6) (p = 0.0026 and p< 0.0001, respectively). Regarding the genera, analyses of median Cqs among *Alouatta* (median Cq = 13.0), *Callicebus* (median Cq = 8.5), and *Callithrix* (median Cq = 32.0) indicated a significant difference (*x*^*2*^ = 31.86, p< 0.0001) (Table 3). Differences in median Cqs were observed for total *Callithrix* compared to *Alouatta* (p< 0.001) and to *Callicebus* (p = 0.0045). Finally, for one specimen of *Sapajus nigritus*, Cq of 30.2 (estimate of 3.10E+04 gc/g liver) was observed.

**Table 3:** Median quantification cycle values and estimates of yellow fever virus genomic load in liver samples of non-human primate carcasses.

Variable	Median Cq (range)	Median YFV genomic copies (range)/ gram of liver*	p-value**
**Environment**			p<0.0001
Urban (n = 130)	32.6 (7.6–37.0)	5.96E+03 (1.74E+11–2.89E+02)	
Urban-rural interface (n = 20)	31.5 (9.2–36.2)	1.27E+04 (5.78E+10–5.01E+02)	
Rural (n = 148)	13.0 (7.7–37)	4.24E+09 (1.62E+11–2.89E+02)	
**Genera**			p<0.0001
*Alouatta* (n = 41)	13.0 (8.2–36.5)	4.24E+09 (1.15E+11–4.08E+02)	
*Callicebus* (n = 22)	8.5 (7.8–36.6)	9.36E+10 (1.51E+11–3.81E+02)	
*Callithrix* (n = 210)	32.0 (8.4–37.0)	9.00E+03 (1.00E+11–2.89E+02)	
**Environment/** **genera**			p<0.0001
Urban *Callithrix* (n = 120)	33.0 (8.9–37)	4.52E+03 (7.11E+10–2.89E+02)	
Urban-rural *Callithrix* (n = 16)	32.8 (9.2–36.2)	5.19E+03 (5.78E+10–5.01E+02)	
Rural *Callithrix* (n = 74)	15.0 (8.4–37)	1.07E+0. (1.00E+11–2.89E+02)	
Rural *Callicebus* (n = 18)	13.9 (7.8–37)	2.28E+09 (1.51E+11–2.89E+02)	
Rural *Alouatta* (n = 33)	14.1 (8.3–36.5)	1.99E+09 (1.07E+11–4.08E+02)	

n: number of carcasses. Cq: quantification cycle value. YFV: yellow fever virus. Liver samples of non-human primate carcasses collected in Minas Gerais state, Brazil (Jan 2017-Dec 2018), were tested for YFV RNA, using the RT-qPCR [24].

*Median Cqs were used to estimate YFV genomic copies per gram of liver using the equation: [10E (-0.2986 x Cq + 13.5094)].

**Median Cqs were compared using the Kruskal-Wallis test with Bonferroni correction for multiple comparisons with differences considered statistically significant at p< 0.0125. Analyses were run in R software v.3.6.0.

When we analyzed median Cqs considering both NHP genera and environments, differences were observed (*x*^*2*^ = 51.58, p< 0.0001) (Table 3). Rural *Alouatta* (median Cq = 14.1), rural *Callicebus* (median Cq = 13.9), and rural *Callithrix* (median Cq = 15.0) presented lower median Cqs (higher estimated YFV genomic loads) compared to urban *Callithrix* (median Cq = 33.0) (p≤ 0.0014). No difference was observed in median Cqs when *Alouatta*, *Callicebus*, and *Callithrix* collected in rural areas were compared to each other (p> 0.20). No difference in median Cqs was observed between urban-rural (median Cq = 33.8) and urban *Callithrix* (p = 0.62).

Partial nucleotide sequences of the envelope gene of 11 samples were obtained (Genbank accession numbers: MN517211-MN517221) (S2 Table), confirming wild-type YFV in NHP carcasses from urban and rural areas of different municipalities of Minas Gerais state. Phylogenetic analysis, involving 112 nucleotide (nt) sequences (621 nt) (S3 Table), indicated that YFV isolates obtained here formed a monophyletic cluster together with other YFV isolates from 2016 to 2018, within South America genotype I (S3 Fig).

## Discussion

From late 2016 to 2019, Southeast Brazil faced large and extensive sylvatic YF outbreaks, with thousands of human cases and epizootics, especially in MG state [4,10–13]. Based on demand for YFV investigation, we received liver samples belonging to NHP carcasses from January/2017 to December/2018. NHP are the primary hosts of the sylvatic YF cycle, and to better understand viral circulation in Minas Gerais state during recent outbreaks, we investigated YFV RNA in NHP carcasses. YFV was detected with high overall positivity of 38% in specimens of *Alouatta*, *Callicebus*, *Sapajus*, and *Callithrix*. We observed the widespread circulation of YFV in Minas Gerais, detecting the virus in 49 municipalities where YF was not previously reported during the recent YF outbreaks [14,25,26]. In Minas Gerais state, the mesoregions with the highest numbers of YFV-positive carcasses were the same ones with the highest records of human YF cases from 2016 to 2018 [4, 14, 23, 24]. Our phylogenetic analyses confirmed the YFV in different urban areas of Minas Gerais, close to human populations. Besides, the YFV detected in urban and rural environments belonged to the same lineage, indicating the dissemination of YFV from rural/sylvatic towards the urban areas during the recent YF outbreaks.

A gradient of YFV dissemination was observed from rural towards urban-rural interface and urban areas, in conformity with the sylvatic pattern of YF [5], when one would expect a higher exposure to the virus in rural environments. According to the seasonal pattern, a higher positivity for YFV infection was observed during the epidemic periods, when a more extensive transmission of the virus and a higher number of cases would likely occur [9–11]. Our data indicate the period from December to March with the highest chances of YFV detection in NHP carcasses, what should be considered by the YF surveillance system, and strengthening the surveillance, especially at the beginning of the epidemic period. Nevertheless, the YFV circulation during the non-epidemic period of 2017 was confirmed here and by official bulletins [10,14] confirming the persistence of YFV in Minas Gerais, from 2017 to 2018 [7], followed by a decline by mid-2018 until 2019 [15].

The NHP genera and species studied here are distributed along with the Atlantic Forest and Cerrado biomes [4,27], the biomes from which NHP carcasses were sampled. *Alouatta* spp. has been described as extremely sensitive to YFV infection, developing severe and fatal disease [1,4]. However, specimens of *Alouatta* presenting antibodies against YFV shows that howler monkeys can survive from YF [4,28,29]. Indeed, high rates of howler monkeys presenting protective or neutralizing antibodies against YFV were described in studies conducted in different regions of Panama (50.7% out of 203 individuals) [29] and Brazil (25.5% out of 192 specimens) [28]. There is little information regarding the susceptibility of *Callicebus* spp. to YFV infection. However, humoral immunity against YFV was demonstrated in 28.6% of 14 specimens investigated by Kumm and Laemmert (1950) [28], in Brazil. In the present study, *Callicebus* and *Alouatta* presented similar proportions of YFV-infected carcasses with similar low median Cqs in RT-qPCR. These results might indicate the high sensitivity to YFV of *Callicebus*, as *C*. *nigrifrons* and *C*. *personatus*. The YF surveillance and animal conservation programs should consider this information since some of those species are threatened (*C*. *nigrifrons*) and vulnerable (*C*. *personatus*) with decreasing population trends [30]. Similarly, species within *Sapajus* genus present population decreasing trends with some already considered critically endangered [30]. Here, only two specimens were sampled, and one carcass was YFV-positive. Although *Sapajus* specimens may develop severe disease, they have been considered less susceptible to YFV [4]. Our results and previous studies [5,7,31] showed YF outbreaks causing thousands of deaths of NHP, which lives in one of the most threatened ecosystems of the world, the Atlantic Forest [4,18].

Despite many marmoset carcasses received (mostly from urban areas), the overall positivity for YFV in *Callithrix* was lower than *Alouatta* and *Callicebus*. Due to their ability to adapt and survive in degraded and secondary habitats [32], free-living marmosets frequently inhabit urban green areas inside Brazilian cities, close to human populations. The proximity of *Callithrix* to the anthropic environment and its wide occurrence in urban areas could increase the chances of dead marmosets to be found in a good preservation status. However, we observed a strong negative correlation for detection of YFV in urban marmosets and higher median Cqs compared to *Callicebus* and *Alouatta*, suggesting lower genomic viral loads in those urban marmosets. Previous studies showed similar data when *Callithrix* presented higher Cqs (lower viral loads) than *Alouatta* during the recent YFV epizootics in Brazil [33]. On the other hand, we did not observe a difference among rural NHP (*Callithrix*, *Alouatta*, and *Callicebus*), which presented median estimates of genomic viral loads of 10E+6 times higher than *Callithrix* from urban and urban-rural transition areas. When only marmosets were evaluated, we detected a higher positivity in rural areas and a decreasing gradient towards urban areas, consistent with the sylvatic pattern of YF. In that way, the lower overall positivity for YFV could result from the lower exposure to YFV-infected sylvatic vectors in urban areas. *Haemagogus* spp. is highly competent and considered the primary vector of YFV in Brazil [1,4,34], including the recent outbreaks in the country [34,35]. These mosquitoes are primatophilic species, being found in the tree canopy [36]. However, some species as *Haemagogus leucocelaenus* and *Haemagogus janthynomis* can disseminate through great distances [34] and may adapt to modified environments, as urban-rural interface [34,35] and urban green areas [37].

In the present study, we observed YFV-positive carcasses collected in urban areas boundaries and different neighborhoods, including downtown areas or near to the central area of different cities. For example, 25-YFV positive marmosets were collected in urban areas of Belo Horizonte (2.5 million inhabitants), the state capital, and a total of 76 YFV-positive marmosets were sampled in the Metropolitan Region of Minas Gerais state (5.0 million inhabitants). Due to some carcasses location (for example, near the downtown area of Belo Horizonte), the land use, and marmosets territorial behavior [32], some of these NHP would have been likely infected in urban matrixes.

Although NHP have a crucial role in the epidemiology of YF and despite YFV-infected NHP in urban areas, there was no documented case of urban/*Aedes* transmission of YFV in Brazil since 1942 [1,4,9–11]. The lower viral load (represented by the estimated viral genomic load) in urban NHP could lower the chances for further viral transmission to vectors. Besides, the closure of some parks in urban centers, the vaccination of humans, and the distinct behavior patterns and habitats observed for hosts (NHP and humans) and vectors in urban areas might have played a role in preventing the urban/*Aedes* transmission of YFV to humans. However, the high infestation of Brazilian urban areas by *Aedes aegypti* poses a risk for YFV urban/*Aedes* transmission [4,34,37,38]. Concerning *Aedes*, previous studies in Brazil have confirmed the competence of *Aedes aegypti* and *Aedes albopictus*, although the latter has shown low transmission competency to YFV [33].

Here we demonstrated the wide occurrence of YFV-infected NHP in Minas Gerais state, including several urban areas, during the epidemic and non-epidemic periods. Although the number of YFV-infected NHP and humans dramatically decreased from the mid-2018, YFV persisted in the Southeast region [5,7,8], at least during three consecutive transmission seasons [39]. These data reinforce that YFV has suitable ecological and climate conditions for its maintenance in Southeast Brazil. Further studies to investigate the sensitivity of urban NHP and the competence of YFV vectors found in urban and urban-rural transition areas should be conducted to understand better the dynamics of YF and the risks for the occurrence of YF in urban centers.

## Supporting information

S1 Fig Characteristics of non-human primate carcasses used for yellow fever virus investigation. (A) Balloon plot of observed frequencies (freq) of NHP carcasses related to the year (2017 and 2018) and the sampling period. Epi: epidemic period (December to May), non-epi: non-epidemic period (June to November). (B) Balloon plot of observed frequencies (freq) of NHP carcasses related to the genera *Alouatta*, *Callicebus*, *Callithrix*, or non-identified specimens (Non-ID), and the environment rural, urban-rural interface (UR-I), or urban areas. Balloon plots were created using Rand; each cell contains a dot whose size and color reflect the relative magnitude of the corresponding component, according to the frequencies presented. (C) Distribution of NHP carcasses according to the epidemiological weeks, in 2017 (left) and 2018 (right). YFV-positive samples are shown in red, and samples non-detectable for YFV RNA by RT-qPCR [24] are shown in blue. Epidemic periods, in 2017 and 2018, correspond to epidemiological weeks 1 to 22 and 49 to 52 of each year.
(TIF)

S2 Fig Association between detection of yellow fever virus (YFV) in the liver of non-human primate carcasses with each bimester within the yellow fever epidemic and non-epidemic periods. YFV ND: yellow fever virus RNA non-detectable. YFV pos: yellow fever virus RNA detectable. Pearson residuals (standardized) were extracted from the chi-square function and plotted. In each cell, the circle’s size is proportional to the amount of cell contribution, and colors indicate positive residuals (blue) or negative residuals (red). The vertical bars indicate Pearson residuals values. Analyses were run in R software v.3.6.0.
(TIF)

S3 Fig Phylogenetic tree of yellow fever virus based on partial envelope gene sequence. The phylogenetic tree was reconstructed using the Maximum Likelihood method based on the General Time Reversible model, using Gamma distribution (5 categories). A total of 1000 bootstrap replicates were run, and circles in the respective nodes represent the results. The analysis involved 112 nucleotide (nt) sequences (621 nt). Branch lengths are drawn to a scale of nucleotide substitutions per site according to the scale. Sequences are identified by Genbank accession. The analysis was conducted in MEGA7, and the final tree was edited and visualized in FigTree v.1.4.4.
(TIF)

S1 Table Non-human primate (NHP) carcasses collected and tested for yellow fever virus (YFV) RNA, according to the mesoregions of Minas Gerais state, Brazil. YFV: yellow fever virus. RT-qPCR: one-step real-time polymerase chain reaction. NHP: non-human primate. Liver samples of NHP carcasses collected in Minas Gerais state, Brazil (January 2017–December 2018), were tested for YFV RNA using the RT-qPCR [24]. (+) detection of YFV RNA by RT-qPCR. (-) non-detection of YFV RNA by RT-qPCR.
(DOC)

S2 Table Information regarding non-human primate (NHP) carcasses from which the yellow fever virus sequences were obtained. ^a^All municipalities are in Minas Gerais state, Southeast of Brazil. NHP: non-human primate. ID: identification. Non-ID: non-identified specimens. Feb.: February. Apr.: April. Jan.: January.
(DOC)

S3 Table Information of yellow fever virus (YFV) sequences included in the dataset. ID: identification. YFV: yellow fever virus. NHP: non-human primate. NA: not available. This dataset included 112 YFV nucleotide (nt) sequences, spanning 621 nt (from the nucleotide 1,766 to 2,386 of MK333808.1 sequence) from South American and African genotypes.
(DOC)
