# Supplementary figures and images for: Neighbor danger: Yellow fever virus epizootics in urban and urban-rural transition areas of Minas Gerais state, during 2017-2018 yellow fever outbreaks in Brazil
Source: PLoS Negl Trop Dis. 2020 Oct 5;14(10):e0008658. doi: 10.1371/journal.pntd.0008658 (PMC7535057; doi:10.1371/journal.pntd.0008658)

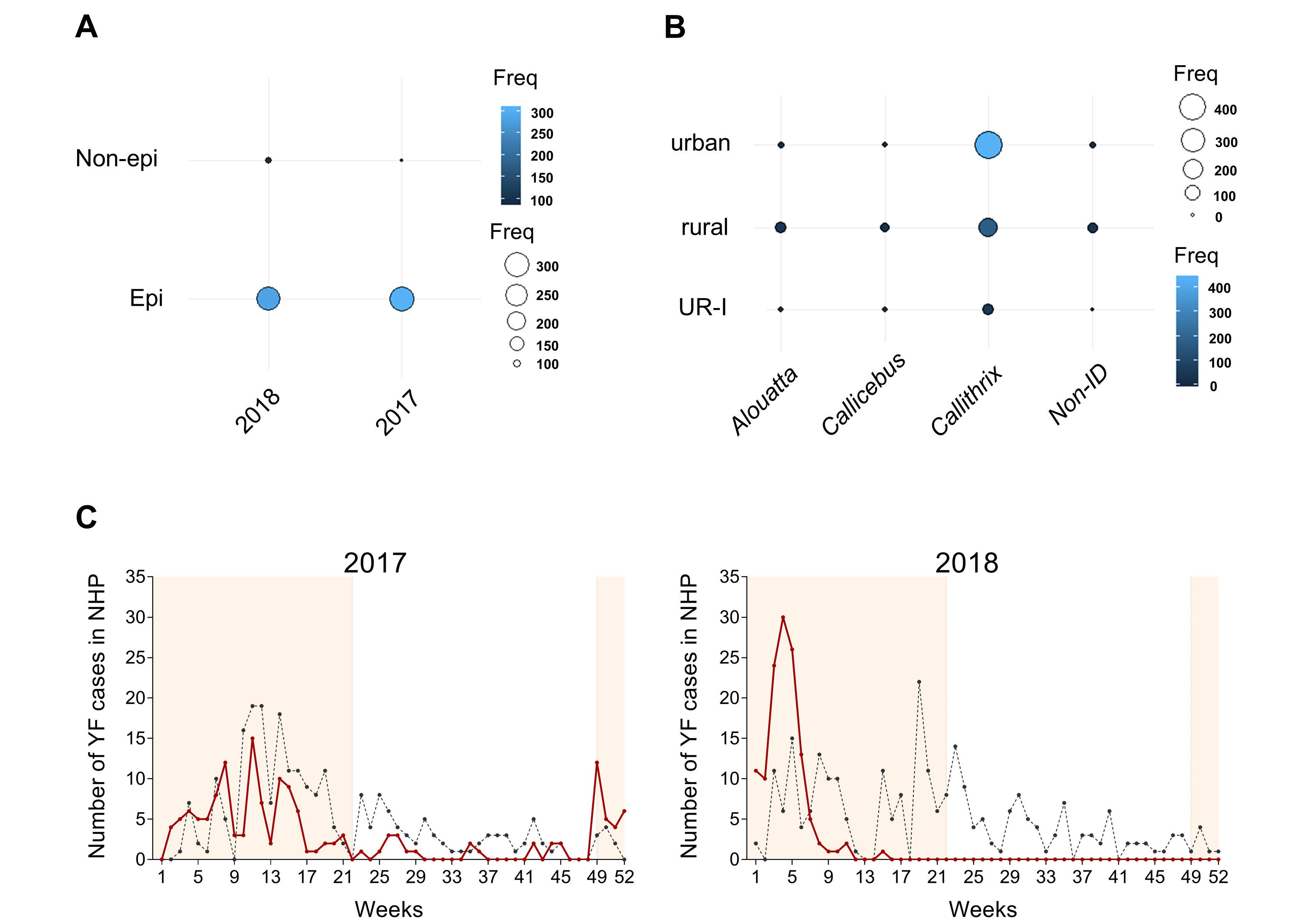

Supplement: S1 Fig — (A) Balloon plot of observed frequencies (freq) of NHP carcasses related to the year (2017 and 2018) and the sampling period. Epi: epidemic period (December to May), non-epi: non-epidemic period (June to November). (B) Balloon plot of observed frequencies (freq) of NHP carcasses related to the genera Alouatta, Callicebus, Callithrix, or non-identified specimens (Non-ID), and the environment rural, urban-rural interface (UR-I), or urban areas. Balloon plots were created using Rand; each cell contains a dot whose size and color reflect the relative magnitude of the corresponding component, according to the frequencies presented. (C) Distribution of NHP carcasses according to the epidemiological weeks, in 2017 (left) and 2018 (right). YFV-positive samples are shown in red, and samples non-detectable for YFV RNA by RT-qPCR [24] are shown in blue. Epidemic periods, in 2017 and 2018, correspond to epidemiological weeks 1 to 22 and 49 to 52 of each year. (TIF) [file pntd.0008658.s001.tif]

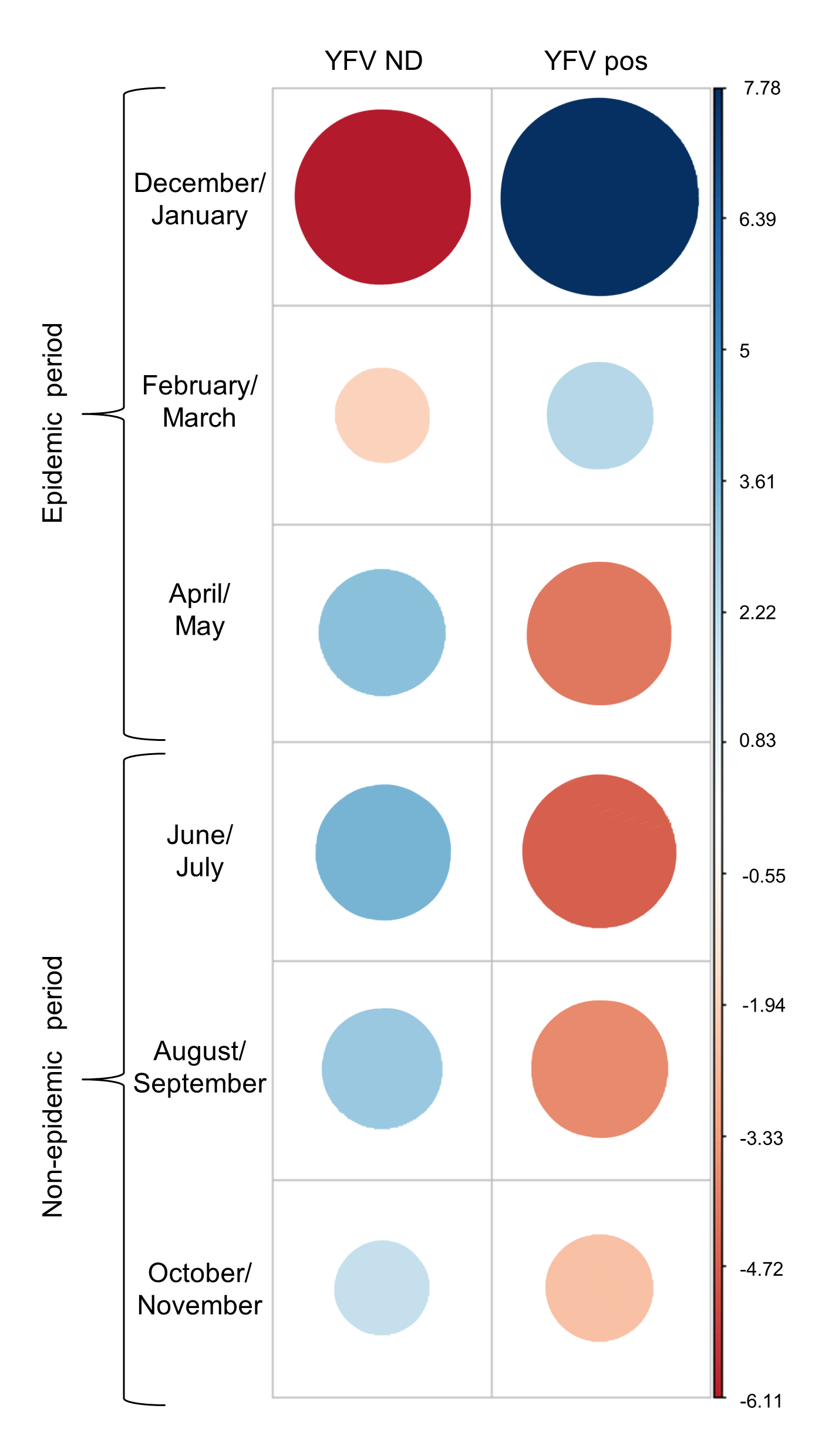

Supplement: S2 Fig — YFV ND: yellow fever virus RNA non-detectable. YFV pos: yellow fever virus RNA detectable. Pearson residuals (standardized) were extracted from the chi-square function and plotted. In each cell, the circle’s size is proportional to the amount of cell contribution, and colors indicate positive residuals (blue) or negative residuals (red). The vertical bars indicate Pearson residuals values. Analyses were run in R software v.3.6.0. (TIF) [file pntd.0008658.s002.tif]

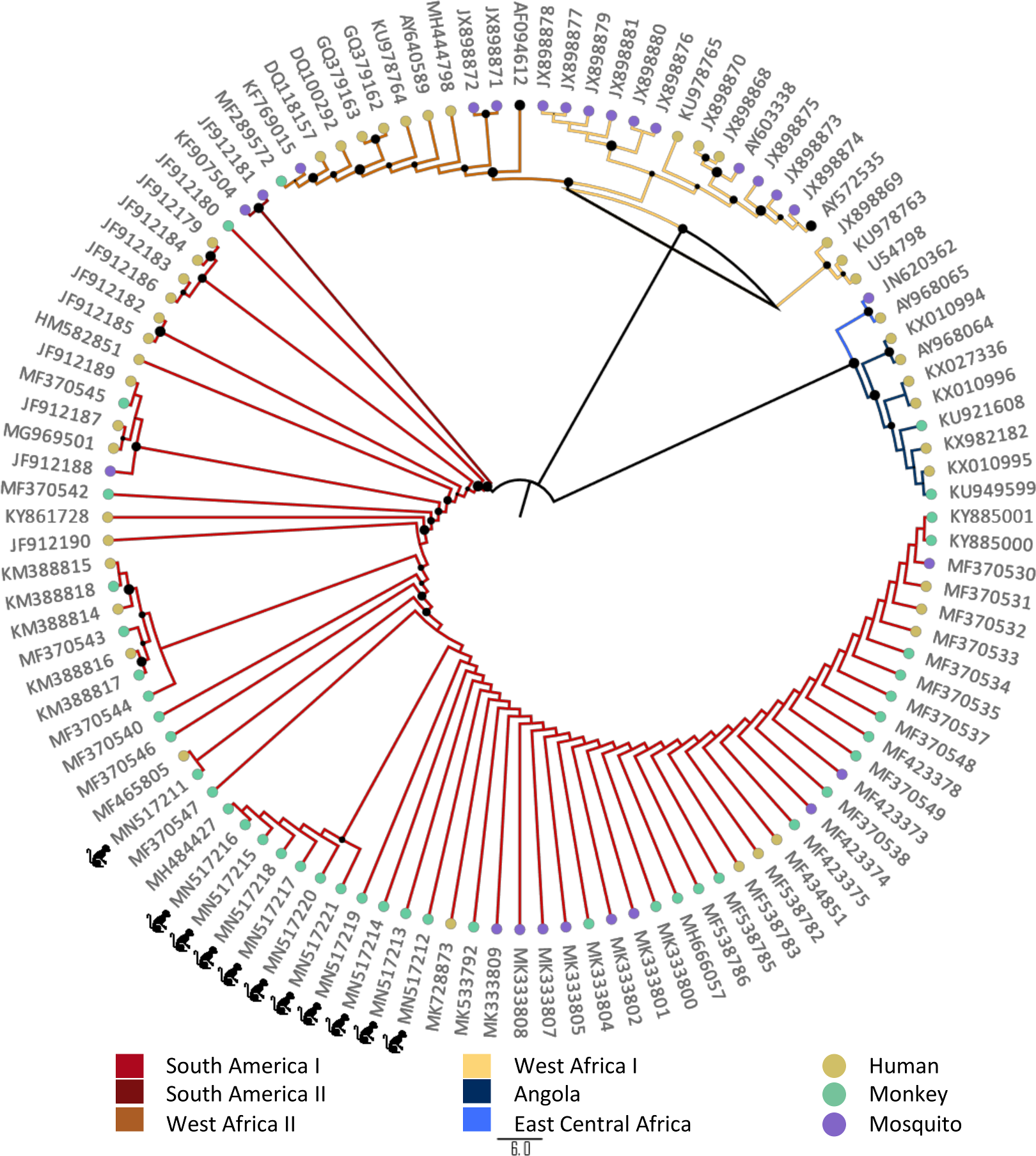

Supplement: S3 Fig — The phylogenetic tree was reconstructed using the Maximum Likelihood method based on the General Time Reversible model, using Gamma distribution (5 categories). A total of 1000 bootstrap replicates were run, and circles in the respective nodes represent the results. The analysis involved 112 nucleotide (nt) sequences (621 nt). Branch lengths are drawn to a scale of nucleotide substitutions per site according to the scale. Sequences are identified by Genbank accession. The analysis was conducted in MEGA7, and the final tree was edited and visualized in FigTree v.1.4.4. (TIF) [file pntd.0008658.s003.tif]
