# Supplementary material for: Neighbor danger: Yellow fever virus epizootics in urban and urban-rural transition areas of Minas Gerais state, during 2017-2018 yellow fever outbreaks in Brazil
Source: PLoS Negl Trop Dis. 2020 Oct 5;14(10):e0008658. doi: 10.1371/journal.pntd.0008658 (PMC7535057; doi:10.1371/journal.pntd.0008658)
Supplement: S1 Table — YFV: yellow fever virus. RT-qPCR: one-step real-time polymerase chain reaction. NHP: non-human primate. Liver samples of NHP carcasses collected in Minas Gerais state, Brazil (January 2017–December 2018), were tested for YFV RNA using the RT-qPCR [24]. (+) detection of YFV RNA by RT-qPCR. (-) non-detection of YFV RNA by RT-qPCR. (DOC) [file pntd.0008658.s004.doc]

S1 Table. Non-human primate (NHP) carcasses collected and tested for yellow fever virus (YFV) RNA, according to the mesoregions of Minas Gerais state, Brazil.

| Mesoregions of Minas Gerais state | YFV RT-qPCR | | | | |
| --- | --- | --- | --- | --- | --- |
|  | 2017 | | 2018 | | YFV-positive NHP carcasses/  total carcasses (%) |
|  | + | - | + | - |  |
| Campo das Vertentes | 8 | 4 | 4 | 5 | 12/21 (57.1%) |
| Central | 4 | 16 | 2 | 12 | 6/36 (17.6%) |
| Jequitinhonha | 0 | 1 | 0 | 6 | 0/7 (0%) |
| Metropolitan | 57 | 120 | 88 | 75 | 145/340 (42.6%) |
| Northwest | 3 | 1 | 3 | 9 | 6/16 (37.5%) |
| North | 4 | 11 | 0 | 5 | 4/20 (20.0%) |
| West | 9 | 5 | 3 | 10 | 12/27 (44.4%) |
| South/Southwest | 28 | 18 | 8 | 28 | 36/82 (43.9) |
| Triângulo/Alto Paranaíba | 6 | 30 | 7 | 51 | 13/94 (12.8%) |
| Vale do Mucuri | 2 | 0 | 0 | 0 | 2/2 (100%) |
| Vale do Rio Doce | 10 | 14 | 2 | 14 | 12/40 (30.0%) |
| Zona da Mata | 24 | 22 | 26 | 26 | 50/90 (51,0%) |
| Total | 155 | 242 | 143 | 241 | 298/781 (38.1%) |

YFV: yellow fever virus. RT-qPCR: one-step real-time polymerase chain reaction. NHP: non-human primate. Liver samples of NHP carcasses collected in Minas Gerais state, Brazil (January 2017–December 2018), were tested for YFV RNA using the RT-qPCR [24]. (+) detection of YFV RNA by RT-qPCR. (-) non-detection of YFV RNA by RT-qPCR.
