# Supplementary material for: Neighbor danger: Yellow fever virus epizootics in urban and urban-rural transition areas of Minas Gerais state, during 2017-2018 yellow fever outbreaks in Brazil
Source: PLoS Negl Trop Dis. 2020 Oct 5;14(10):e0008658. doi: 10.1371/journal.pntd.0008658 (PMC7535057; doi:10.1371/journal.pntd.0008658)
Supplement: S2 Table — aAll municipalities are in Minas Gerais state, Southeast of Brazil. NHP: non-human primate. ID: identification. Non-ID: non-identified specimens. Feb.: February. Apr.: April. Jan.: January. (DOC) [file pntd.0008658.s005.doc]

S2 Table. Information regarding non-human primate (NHP) carcasses from which the yellow fever virus sequences were obtained.

| Samples | GenBank ID | Date | Genera | Area | Municipalitya |
| --- | --- | --- | --- | --- | --- |
| NHP31 | MN517211 | Feb/17/17 | Alouatta | Urban | Santa Rita de Caldas |
| NHP179 | MN517212 | Apr/06//17 | Callithrix | Urban | Betim |
| NHP237 | MN517213 | May/05/17 | Alouatta | Rural | Rio Doce |
| NHP265 | MN517214 | Apr/07/17 | Callithrix | Urban | São Tiago |
| NHP450 | MN517215 | Jan/01/18 | Non-ID | Rural | Nova Lima |
| NHP481 | MN517216 | Jan/10/18 | Callithrix | Rural | Brumadinho |
| NHP525 | MN517217 | Jan/16/18 | Callithrix | Rural | Bonfim |
| NHP354 | MN517218 | Jan/18/18 | Callithrix | Urban | Barão de Cocais |
| NHP541 | MN517219 | Jan/30/18 | Callithrix | Rural | Santa Bárbara |
| NHP637 | MN517220 | Feb/05/18 | Callithrix | Urban | Itabirito |
| NHP657 | MN517221 | Feb/01/18 | Callithrix | Urban | Itabirito |

aAll municipalities are in Minas Gerais state, Southeast of Brazil. NHP: non-human primate. ID: identification. Non-ID: non-identified specimens. Feb.: February. Apr.: April. Jan.: January.
