# Supplementary material for: Neighbor danger: Yellow fever virus epizootics in urban and urban-rural transition areas of Minas Gerais state, during 2017-2018 yellow fever outbreaks in Brazil
Source: PLoS Negl Trop Dis. 2020 Oct 5;14(10):e0008658. doi: 10.1371/journal.pntd.0008658 (PMC7535057; doi:10.1371/journal.pntd.0008658)
Supplement: S3 Table — ID: identification. YFV: yellow fever virus. NHP: non-human primate. NA: not available. This dataset included 112 YFV nucleotide (nt) sequences, spanning 621 nt (from the nucleotide 1,766 to 2,386 of MK333808.1 sequence) from South American and African genotypes. (DOC) [file pntd.0008658.s006.doc]

**S3 Table. Information of yellow fever virus (YFV) sequences included in the** dataset.

| **GenBank ID** | **YFV strain** | **Origin** | **Year** |
| --- | --- | --- | --- |
| MN517211 | NHP31 | Brazil | 2017 |
| MN517212 | NHP179 | Brazil | 2017 |
| MN517213 | NHP237 | Brazil | 2017 |
| MN517214 | NHP265 | Brazil | 2017 |
| MN517215 | NHP450 | Brazil | 2018 |
| MN517216 | NHP481 | Brazil | 2018 |
| MN517217 | NHP525 | Brazil | 2018 |
| MN517218 | NHP354 | Brazil | 2018 |
| MN517219 | NHP541 | Brazil | 2018 |
| MN517220 | NHP637 | Brazil | 2018 |
| MN517221 | NHP657 | Brazil | 2018 |
| MK728873.1 | GO21 | Brazil | 2017 |
| MK533792.1 | RJ 155 | Brazil | 2019 |
| MK333809.1 | VL2926 | Brazil | 2018 |
| MK333808.1 | MG3155 | Brazil | 2018 |
| MK333807.1 | MG3121 | Brazil | 2018 |
| MK333805.1 | IG3036 | Brazil | 2018 |
| MK333804.1 | GO27 | Brazil | 2015 |
| MK333802.1 | ES2750 | Brazil | 2017 |
| MK333801.1 | ES2682 | Brazil | 2017 |
| MK333800.1 | ES03 | Brazil | 2017 |
| MH666057.1 | SPAn 14/2017 | Brazil | 2017 |
| MH484427.1 | YFV/Monkey/Brazil/2017/RJ153 | Brazil | 2017 |
| U54798.1 | 85-82H Ivory Coast | Ivory Coast | 1982 |
| MH444798.1 | FVV | Senegal | 1927 |
| MG969501.1 | FIOCRUZ 71530/MG/01 | Brazil | 2001 |
| MF538786.2 | RJ104 | Brazil | 2017 |
| MF538785.2 | RJ97 | Brazil | 2017 |
| MF538783.2 | H191 | Brazil | 2017 |
| MF538782.2 | H190 | Brazil | 2017 |
| MF465805.1 | YFV112 | Brazil | 2017 |
| MF434851.2 | H199 | Brazil | 2017 |
| MF423378.2 | RJ96 | Brazil | 2017 |
| MF423375.2 | RJ87 | Brazil | 2017 |
| MF423374.2 | PA196 | Brazil | 2017 |
| MF423373.2 | PA193 | Brazil | 2017 |
| MF370549.1 | BeAn820890 | Brazil | 2015 |
| MF370548.1 | RJPR4867 | Brazil | 2017 |
| MF370547.1 | RJPR2910 | Brazil | 2017 |
| MF370538.1 | PR5873-BeAn843840 | Brazil | 2017 |
| MF370537.1 | PR5841-BeAn843345 | Brazil | 2017 |
| MF370535.1 | PR5818-BeAn841176 | Brazil | 2016 |
| MF370534.1 | PR5782-BeAn838691 | Brazil | 2016 |
| MF370533.1 | MIG2484-H843301 | Brazil | 2017 |
| MF370532.1 | MIG2482-H843297 | Brazil | 2017 |
| MF370531.1 | ESS1115-H844801 | Brazil | 2017 |
| MF370530.1 | BeAr843721 | Brazil | 2017 |
| KY885001.2 | ES-505 | Brazil | 2017 |
| KY885000.2 | ES-504 | Brazil | 2017 |
| MF370545.1 | PR5952-BeAn845405 | Brazil | 2017 |
| JF912186.1 | BeH526722 | Brazil | 1994 |
| MF370546.1 | PR5953-BeAn845409 | Brazil | 2017 |
| MF370544.1 | PR5951-BeAn845401 | Brazil | 2017 |
| MF370542.1 | PR5926-BeAn844893 | Brazil | 2017 |
| MF370543.1 | PR5937-BeAn845130 | Brazil | 2017 |
| MF370540.1 | PR5892-BeAn844358 | Brazil | 2017 |
| MF289572.1 | YFV_EHI | Singapore | 2017 |
| KY861728.1 | BeAn754036 (PR4408) | Brazil | 2008 |
| KX982182.1 | Cahama-C17 | Angola | 2016 |
| KX027336.1 | CIC4 | China | 2016 |
| KX010996.1 | CIC3 | China | 2016 |
| KX010995.1 | CIC2 | China | 2016 |
| KX010994.1 | CIC1 | China | 2016 |
| KU978765.1 | Jose Cachatra | Guinea-Bissau | 1965 |
| KU978764.1 | M 185 D 160 | Sudan | 1941 |
| KU978763.1 | Ogbomosho | Nigeria | 1946 |
| KU949599.1 | Shanghai01 | China | 2016 |
| KU921608.1 | CNYF01/2016 | China | 2016 |
| KM388818.1 | 8A | Venezuela | 2006 |
| KM388817.1 | 2A | Venezuela | 2004 |
| KM388816.1 | 10A | Venezuela | 2010 |
| KM388815.1 | 9A | Venezuela | 2007 |
| KM388814.1 | 6A | Venezuela | 2005 |
| KF907504.1 | 88/1999 | Bolivia | 1999 |
| KF769015.1 | 17D-204 | United States | NA |
| JX898881.1 | ArD181439 | Senegal | 2005 |
| JX898880.1 | ArD181564 | Senegal | 2005 |
| JX898879.1 | ArD181676 | Senegal | 2005 |
| JX898878.1 | ArD181250 | Senegal | 2005 |
| JX898877.1 | ArD181464 | Senegal | 2005 |
| JX898876.1 | ArD156468 | Senegal | 2001 |
| JX898875.1 | ArD149815 | Senegal | 2000 |
| JX898874.1 | ArD149194 | Senegal | 2000 |
| JX898873.1 | ArD149214 | Senegal | 2000 |
| JX898872.1 | ArD114972 | Senegal | 1995 |
| JX898871.1 | ArD114896 | Senegal | 1995 |
| JX898870.1 | ArD121040 | Senegal | 1996 |
| JX898869.1 | DakArAmt7 | Cote d'Ivoire | 1973 |
| JX898868.1 | HD117294 | Senegal | 1995 |
| JN620362.1 | Uganda 2010 | Uganda | 2010 |
| JF912190.1 | BeH655417 | Brazil | 2002 |
| JF912189.1 | BeAR646536 | Brazil | 2001 |
| JF912188.1 | BeH622493 | Brazil | 2000 |
| JF912187.1 | BeH622205 | Brazil | 2000 |
| JF912185.1 | BeAR513008 | Brazil | 1992 |
| JF912184.1 | BeH463676 | Brazil | 1987 |
| JF912183.1 | BeH423602 | Brazil | 1984 |
| JF912182.1 | BeH422973 | Brazil | 1984 |
| JF912181.1 | BeH413820 | Brazil | 1983 |
| JF912180.1 | BeH394880 | Brazil | 1981 |
| JF912179.1 | BeAR378600 | Brazil | 1980 |
| HM582851.1 | TVP11767 | Trinidad and Tobago | 2009 |
| GQ379163.1 | case #2 | Peru | 2007 |
| GQ379162.1 | case #1 | Peru | 2007 |
| DQ118157.1 | YF-AVD2791-93F/04 | Spain | 2004 |
| AY968065.1 | Uganda48a | Uganda | 1948 |
| AY968064.1 | Angola71 | Angola | 1971 |
| DQ100292.1 | 17DD-Brazil | Brazil | NA |
| AY640589.1 | ASIBI | Ghana | 1927 |
| AY603338.1 | Ivory Coast 1999 | Cote d'Ivoire | 1999 |
| AY572535.1 | Gambia 2001 | Gambia | 2001 |
| AF094612.1 | Trinidad 79A | Trinidad and Tobago | 1979 |

ID: identification. YFV: yellow fever virus. NHP: non-human primate. NA: not available. This dataset included 112 YFV nucleotide (nt) sequences, spanning 621 nt (from the nucleotide 1,766 to 2386 of MK333808.1 sequence) from South American and African genotypes.
